# Challenges of Fertility Sparing Ovarian Surgery Imposed by Krukenberg Tumors in Pregnancy

**DOI:** 10.23937/2378-3656/1410041

**Published:** 2015-06-08

**Authors:** Michael H. Bloch, Ramey Z. Elsarrag, Mazin Z. Elsarrag, Sana M. Salih

**Affiliations:** 1 Child Study Center, Department of Psychiatry, Yale University School of Medicine, USA; 2 Department of Obstetrics and Gynecology, Divisions of Reproductive Endocrinology and Infertility, University of Wisconsin, USA

**Keywords:** ovary, krukenberg tumor, steroid cell tumor, pregnancy, fertility preservation, fertility sparing surgery, frozen section

## Abstract

Fertility sparing surgery is advocated for reproductive-age women with benign and borderline ovarian tumors. The hormonal milieu of pregnancy may, however, complicate the decision making process. The patient presented in the third trimester with a rapidly growing tumor that was diagnosed as benign steroid cell tumor by intraoperative frozen section. Fertility-sparing surgery with right oophorectomy and partial left oophorectomy was performed. The final pathology examination demonstrated signet cells staining positive for mucin, which is pathognomonic for Krukenberg tumors. Krukenberg cells were overlooked in the frozen section due to the predominance of hormonally active luteinized ovarian stroma cells. This case highlights the challenges associated with fertility sparing surgery in women presenting with ovarian tumors in pregnancy and the limitations of frozen section in providing an accurate diagnosis.

## Introduction

Gynecologic tumors are rare and account for approximately 3% of all cancers in reproductive-aged women [1]. Fertility-sparing surgery (FSS) with ovarian cystectomy and/or unilateral oophorectomy is the major objective in reproductive-age women diagnosed with ovarian tumors whom still desire to preserve fertility. FSS is advocated for early-stage borderline ovarian tumors, even when the tumor is bilateral or recurrent [2]. FSS is also advocated for early epithelial ovarian cancers, but there is no consensus as to the grade of the tumor that is eligible (FIGO stage 1, grade 1 and grade 2 in highly selected patients, although grade 3 has been considered) [3]. With preponderance of steroid cell tumors in young adults, FSS is advocated for tumors that lack malignant features [4,5]. Despite the wide acceptance of FSS, tumor-specific guidelines and the safety profiles of FSS are not clear. Most reports about FFS are encouraging, but others carry a cautionary note [6,7]. The balance between the risk of cancer recurrence and ovarian preservation by FSS is delicate and complicated by the inability to accurately predict the long-term malignant potential of the tumor.

Krukenberg tumors occurring during pregnancy are extremely rare in the Western World [8]. The challenge in diagnosing a Krukenberg tumor in pregnancy is understandable, given the expanding uterus’ ability to camouflage the tumor and the symptoms of pregnancy, which mimic the presenting symptoms of gastric cancer. We describe a case of a Krukenberg tumor that presents as a steroid cell tumor in pregnancy and discuss the management options of this rare tumor in pregnancy.

## Case

**Figure 1:**
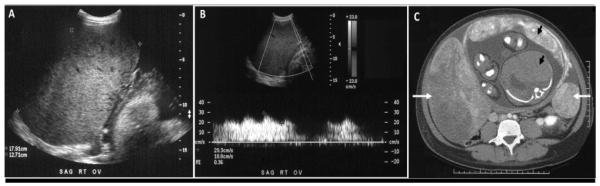
***A.*** Transbdominal ultrasound image of the right ovary showing an enlarged heterogeneous complex mass measuring 13.0×18.7×11.0cm with moderate ascites. ***B.*** Doppler ultrasound of the right ovarian mass at 35 weeks gestation showing abnormal Doppler signals, with a peak systolic velocity of 34.5cm per second, end diastolic velocity of 21.7cm per second, and a resistive index of 0.37, suggestive of neovascularization and decreased impedance to blood flow. ***C.*** Contrast-enhanced computed tomography scan of the abdomen at 35 weeks gestation showing bilateral ovarian masses (white arrows) and a cross section of the uterus, placenta, and fetus (black arrows).

A 27 year-old Hispanic female presented at 35 weeks gestation with a recent onset of lower quadrant abdominal pain and a rapidly increasing abdominal girth over a one-week period. At 35 weeks gestation her fundal height had increased to 37cm and her flanks were distended with a positive ascitic fluid wave. A transabdominal ultrasound revealed two large adnexal masses with both solid and cystic components and moderate ascites. The right adnexal mass measured 13.0×18.7×11.0cm, while the left adnexal mass measured 5.2×6.0×4.5cm (Figure 1A). There was increased blood flow to both ovaries, with a reduced resistant index of 0.31 (Figure 1B). An abdominal computed tomography scan confirmed bilateral ovarian masses (Figure 1C) and revealed no evidence of intraperitoneal metastasis or omental caking. Tumor markers were drawn to rule out hormonally active ovarian tumors. CA-125 was elevated at 698U/mL (mean level during pregnancy is 204U/ml at term). Her serum testosterone was <16ng/ml (normal 0.1–0.9ng/ml), percent free testosterone was slightly elevated at 49% (normal 25%-35%), and free testosterone <8.0ng/ml (normal 0.002–0.2ng/ml). The remainder of the hormone profile included normal CA19-9, CEA, and Inhibin.

**Figure 2:**
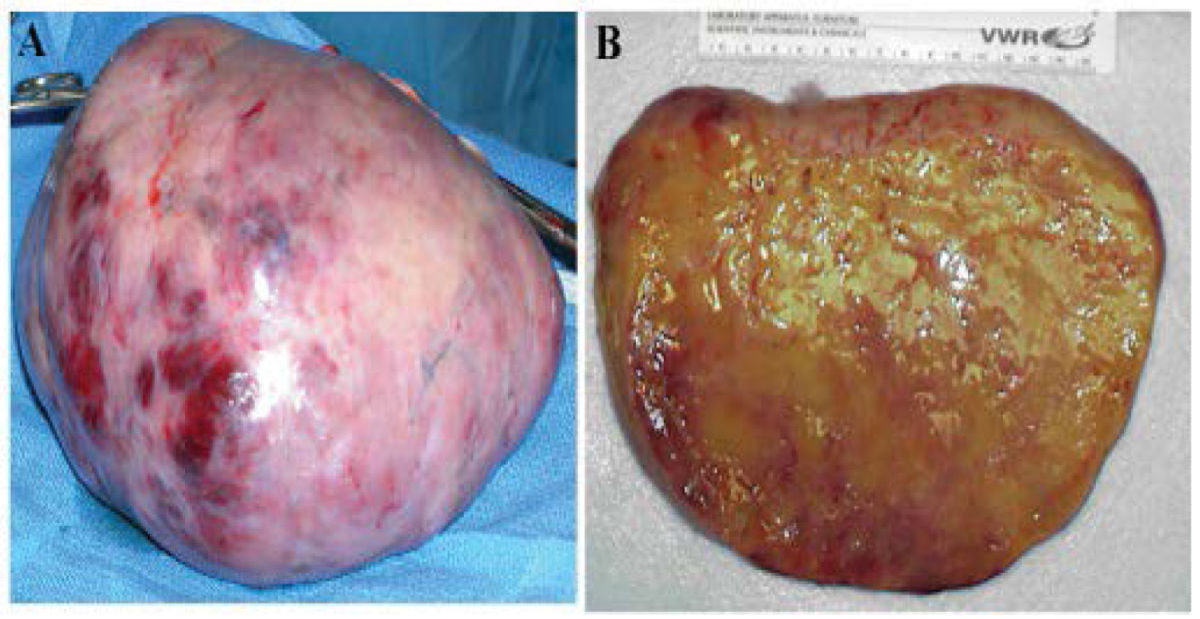
***A.*** The right ovary replaced by a 20cm tumor mass, which weighed 2400g. ***B.*** The mass has a homogenous, yellow, soft consistency resembling steroid cell tumor or a pregnancy luteoma.

An ultrasound-guided paracentesis drained 250μL of serous fluid, which was negative for malignant cells. A preliminary diagnosis of ovarian neoplasm of unknown etiology and the decision to perform exploratory laparotomy to biopsy, debulk, and stage the ovarian tumor in the postpartum period was made. Within a couple of days of admission, the patient delivered a healthy female baby vaginally with no evidence of neonatal virilization. In the immediate postpartum period, the patient’s abdomen grew increasingly tense and distended and she developed intermittent ovarian torsion. An emergency exploratory laparotomy revealed three liters of ascitic fluid. A right oophorectomy yielded a 20 cm ovarian mass, which traversed the entire ovary. The right ovary weighed 2400g and had a soft, yellow consistency when cut (Figure 2A,2B). The mass was homogenous with no cystic lesions, but some areas of infarction were present. The intraoperative frozen section showed steroid producing cells, most likely a steroid cell tumor, but possibly bilateral luteomas. Since the vast majority of steroid cell tumors have a low-grade malignant potential, the left ovarian mass was resected, but any remaining viable ovarian tissue was preserved, in order to preserve fertility as desired by the patient. There were no enlarged lymph nodes. An infracolic omentectomy was performed and no gross evidence of metastatic disease was present.

**Figure 3:**
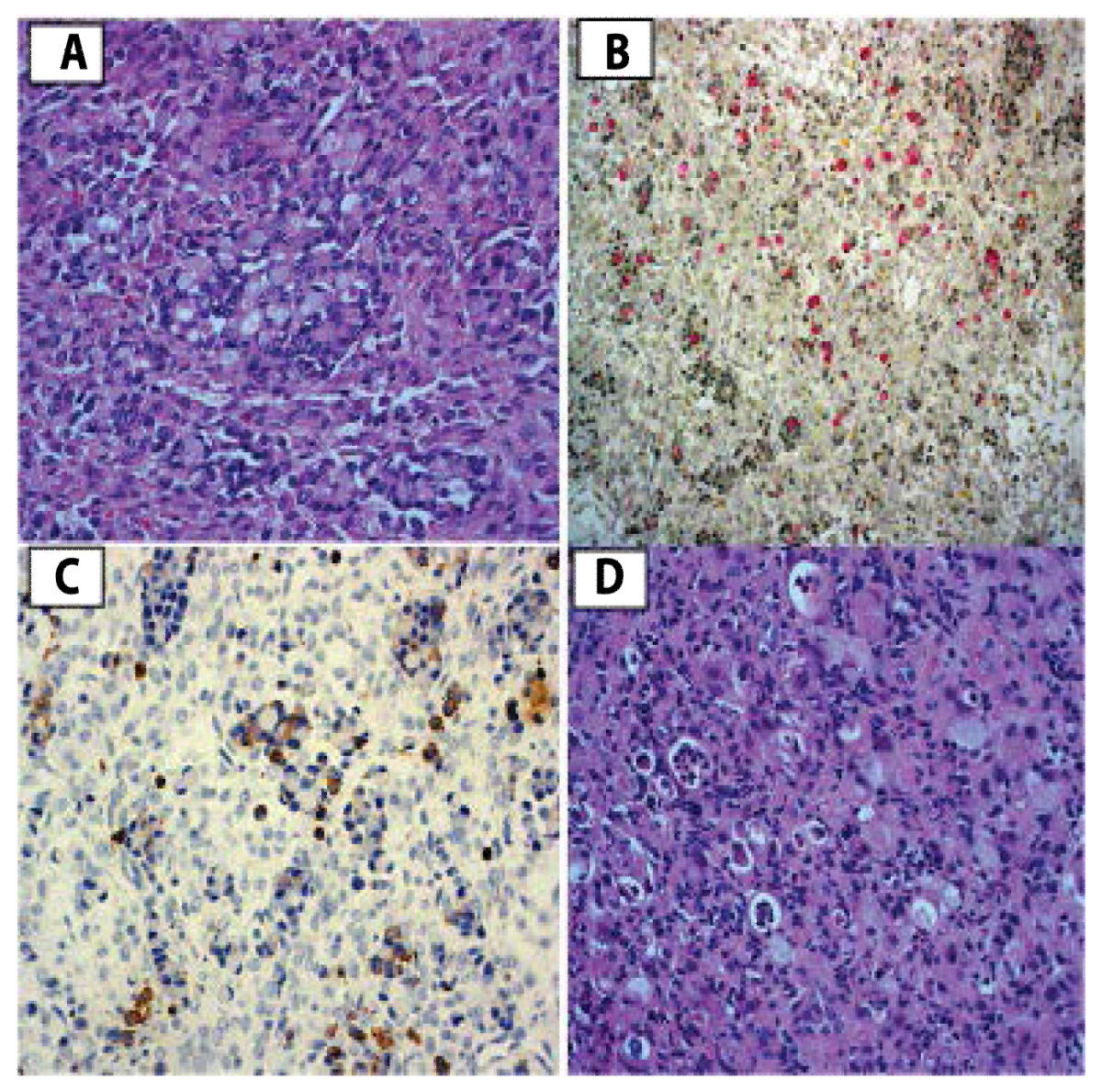
***A.*** Ovary: Signet ring cells infilterating the ovary, pathognomic of Krukenberg tumor of the ovary (H&E). ***B.*** Ovary: The cells stained positive for mucicarmine stain indicating true mucin secretion. ***C.*** Ovary: Histoimmunostaining positive for Cytokeratin, indicating the epithelial origin of the tumor and excluding steroid cell tumor. ***D.*** Stomach biopsy revealed similar signet ring cells consistent with primary gastric adenocarcinoma (H&E). for all images.

Permanent sections of pathology tissue, however, demonstrated signet-ring cells of epithelial origin, staining positive for mucin and cytokeratin (Figure 3A-3C) consistent with Krukenberg’s tumor. The ascitic fluid and the omentum were negative for malignancy. An interval upper gastroendoscopy revealed poorly differentiated signet-ring cells located in the cardiac region of the stomach, confirming the gastric origin of the Krukenberg tumor (Figure 3D). The patient was started on Oxaliplatin, 5-Florouracil, and Calcium Leucovorin chemotherapy. She was discharged home in stable condition on the 6^th^ postoperative day. Nine months after surgery after surgery the patient remains in partial remission, but subsequently developed cancer progression, necessitating further gastric surgery and chemotherapy. Her daughter remained well and healthy.

## Discussion

This case highlights the complex decision-making process that is needed in order to offer FSS to preserve fertility in reproductive-age women diagnosed with ovarian tumors during pregnancy. It illustrates the pitfalls of frozen section to provide an accurate intraoperative diagnosis, which might affect the decision of treatment at the time of surgery. A Krukenberg tumor is broadly defined as an adenocarcinoma metastatic to the ovary. Gastric carcinoma, particularly with concomitant Krukenberg tumors, carries a dire prognosis. The median survival time from diagnosis to death in all comers is approximately one year only [9]. This tragic prognosis is attributable to the aggressive growth characteristics of gastric carcinoma and the relative paucity of symptoms until widely metastatic disease is present [10]. Krukenberg tumors discovered in pregnancy or in the immediate postpartum period carry an even more daunting prognosis, even when the survival rates are compared to Krukenberg tumors arising in an age and gender matched population [8,11]. The especially poor prognosis attributed to Krukenberg tumors arising in pregnancy has been attributed to the presence of estrogen receptors in a high proportion of gastric tumors, and an increased proportion of tumors with high-grade pathology arising in pregnant women [12]. Krukenberg tumors mimicking pregnancy luteomas and associated with virilization and massive luteinization of the ovary have been reported [13,14]. It is possible that, the tumor cells may stimulate the ovarian stroma to differentiate into hormonally active cells (luteinized stroma cells), thereby inducing androgen or estrogen, thus mimicking hormonally active primary ovarian tumors such as Sertoli-Leydig cell tumors, steroid cell tumors, or pregnancy luteomas.

The patient presented late in the third trimester with ascites and bilateral ovarian masses. Steroid cell tumors often present as unilateral solid tumors and occasionally as cystic tumors, usually with necrosis or calcification [4]. The delay in diagnosis of gastric cancer during pregnancy is cofounded by mitigation of the symptoms of gastric cancer or ovarian metastases by pregnancy. Symptomatic gastric carcinoma typically presents with vomiting, bloating, epigastric pain and/or weight loss. The potent ability of the normal physiology of pregnancy to explain away worrisome symptoms of gastric carcinoma is evidenced by examining the frequency of emesis during pregnancy. If all women with significant emesis underwent gastroscopy to exclude gastric carcinoma, an estimated 1,000,000 such procedures would need to be performed to diagnose just nine cases of gastric carcinoma [15]. Further impeding gastric cancer diagnosis in pregnancy is the hesitancy of doctors and patients to undergo radiographic procedures, such as abdominal CT or barium ingestion, during the gestation because of the feared teratogenic effects of radiation. The role of tumor markers such as CA125 and Inhibin remain unproven even for the management of nonpregnant women with ovarian malignancies. The use of these markers in pregnancy is even more problematic.

FSS is complicated by the need to accurately diagnose the tumor prior to conservative ovarian surgery. Age is an important factor for the differential diagnosis of primary and secondary ovarian tumors. Additionally, many operative findings can raise the suspicion of malignancy. Metastatic ovarian tumors from the breast, colon and stomach are typically bilateral and are associated with ascites and omenal involvement [10] . This is in contrast to a steroid-producing tumor of the ovary, which are unilateral in 94% of cases, and are not associated with ascites [4]. Despite the high index of suspicion for malignancy in our case based on the bilateral position of the tumor and the presence of ascites, the frozen section returned as a benign steroid cell tumor. Although debulking surgery of metastatic tumor of the ovary is seldom considered curative, recent studies showed ovarian metastasectomy prolongs disease free survival in selective cases [10]. Frozen section is known for its pitfall, particularly when it comes to metastatic tumors. Improved imaging, molecular diagnostic modalities, and molecular pathology prior to surgery are predicted to achieve a better understanding of the malignant nature of ovarian tumors.

In this case, the pathology of fixed tissue revealed a Krukenberg tumor. Optimal management of Krukenberg tumors with a gastric primary includes partial or total gastrectomy, lymph node dissection, bilateral oophorectomy, and peritoneal washings. The postoperative course includes chemotherapy with cisplatin and 5-Fluorouracil or capecitabine and oxaliplatin [17]. Treatment of known Krukenberg tumors during pregnancy raises difficult questions for both patient and physician, since both the life of the mother and the fetus need to be considered, especially for Krukenberg tumors discovered in the third trimester of pregnancy. Optimal management should involve expedited delivery of the child, either through premature induction of labor or cesarean section, followed by surgical treatment of the tumor as soon as the mother has recovered from the stress of delivery. An operative cesarean section can be combined with surgical resection of the tumor when this is deemed the most prudent course of therapy. Chemotherapy is dictated by the origin of the tumor and should be initiated in the postpartum period and the newborn must be bottle-fed throughout the postpartum period.

FSS is contraindicated in patients with Krukenberg tumor. Considering the poor prognosis of Krukenberg tumors in general, however, the said surgery might not have changed her prognosis. Deciphering the correct ovarian tumor for FSS will always remain a challenge and only time will tell whether the right decision was made to preserve fertility in any particular patient.
